# Cross-regulation between Aurora B and Citron kinase controls midbody architecture in cytokinesis

**DOI:** 10.1098/rsob.160019

**Published:** 2016-03-23

**Authors:** Callum McKenzie, Zuni I. Bassi, Janusz Debski, Marco Gottardo, Giuliano Callaini, Michal Dadlez, Pier Paolo D'Avino

**Affiliations:** 1 Department of Pathology, University of Cambridge, Tennis Court Road, Cambridge CB2 1QP, UK; 2 Mass Spectrometry Laboratory, Institute of Biochemistry and Biophysics, Warszawa 02-106, Poland; 3 Department of Life Sciences, University of Siena, Via A. Moro 4, Siena 53100, Italy

**Keywords:** Aurora B, Citron kinase, midbody, cell division

## Abstract

Cytokinesis culminates in the final separation, or abscission, of the two daughter cells at the end of cell division. Abscission relies on an organelle, the midbody, which forms at the intercellular bridge and is composed of various proteins arranged in a precise stereotypic pattern. The molecular mechanisms controlling midbody organization and function, however, are obscure. Here we show that proper midbody architecture requires cross-regulation between two cell division kinases, Citron kinase (CIT-K) and Aurora B, the kinase component of the chromosomal passenger complex (CPC). CIT-K interacts directly with three CPC components and is required for proper midbody architecture and the orderly arrangement of midbody proteins, including the CPC. In addition, we show that CIT-K promotes Aurora B activity through phosphorylation of the INCENP CPC subunit at the TSS motif. In turn, Aurora B controls CIT-K localization and association with its central spindle partners through phosphorylation of CIT-K's coiled coil domain. Our results identify, for the first time, a cross-regulatory mechanism between two kinases during cytokinesis, which is crucial for establishing the stereotyped organization of midbody proteins.

## 1. Introduction

**Figure 1.**
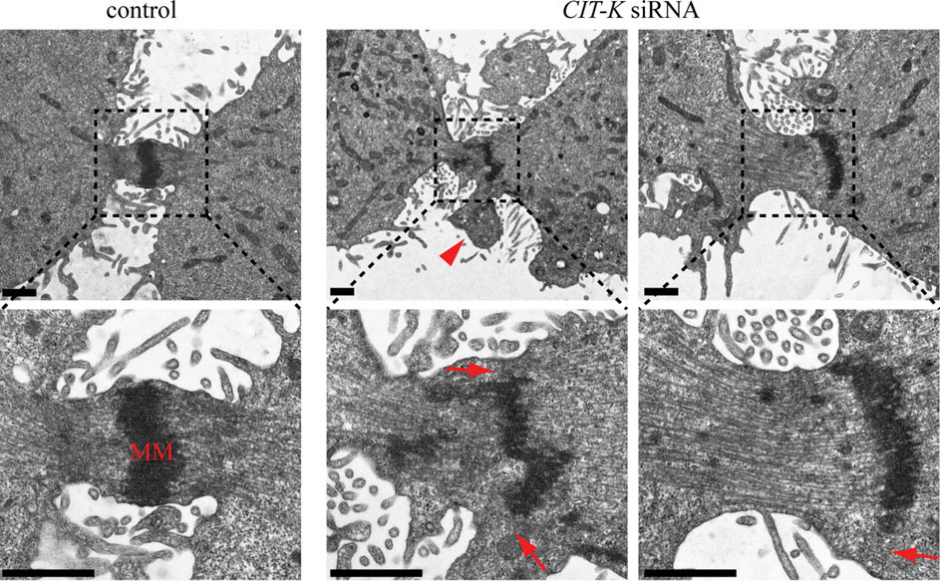
CIT-K is required for midbody architecture. EM images of midbodies in HeLa Kyoto cells treated with an siRNA directed against either a random sequence (control) or *CIT-K* for 48 h. The bottom panels are magnification of the insets as indicated by dotted lines. The arrowhead marks a membrane bleb and the arrows indicate gaps between the midbody matrix and the cortex. MM, midbody matrix. Scale bars, 1 µm.

Cytokinesis controls the proper partitioning of cytoplasmic and nuclear material between the two nascent daughter cells. Defects in this process have been associated with various human diseases [1] and can cause polyploidy, which in turn can lead to chromosomal instability, a hallmark of cancer cells [2]. Cytokinesis progresses through a series of sequential events. First, after anaphase onset the mitotic spindle is completely reorganized into an array of interdigitating and antiparallel microtubules, known as the central spindle [3]. These microtubules are tightly bundled at their plus ends in a region known as the spindle midzone. Signals from astral and central spindle microtubules promote the formation of a cleavage furrow at the cell's equator that ingresses to bisect the mother cell [4]. Furrow ingression is driven by the assembly and constriction of an actomyosin contractile ring, which progressively compacts the central spindle to form an organelle known as the midbody. The midbody provides a platform necessary for the recruitment and organization of many proteins that regulate the final abscission of the two daughter cells [5] and it has also been proposed to contribute to cell fate determination [6,7], albeit this has not been confirmed in intact organisms [8,9]. The midbody contains at its centre an electron dense structure known as the ‘midbody matrix’ or ‘midbody ring’ (figure 1). The regions flanking the midbody ring are often indicated as ‘midbody arms’. Some midbody proteins are former contractile ring and central spindle components, while others are recruited after completion of furrow ingression. All these proteins show a very precise and stereotypic distribution along the midbody [10]. For example, some contractile ring and midzone components, such as Citron kinase (CIT-K) and the kinesin KIF23/MKLP1, accumulate at the midbody ring, while other central spindle proteins, such as the chromosomal passenger complex (CPC), localize to the midbody arms. This precise distribution pattern is necessary for midbody assembly and function, but what controls the architecture of the midbody is unclear. CIT-K is emerging as an important midbody organizer. This kinase links a network of contractile ring and central spindle proteins—including actin, Anillin, myosin, KIF23, KIF14 and RhoA—in both *Drosophila* and human cells and has also recently been shown to be important for the stability of midbody microtubules in some mouse tissues [11–16]. CIT-K was initially identified as a RhoA effector and proposed to promote contractile ring constriction through phosphorylation of the myosin regulatory light chain (MRLC) [17,18]. However, CIT-K and its *Drosophila* orthologue, Sticky (Sti), are not required for furrowing [13,19–22] and evidence in both *Drosophila* and human cells has shown that CIT-K is required for proper RhoA localization at the cleavage site during late cytokinesis, thus behaving more like a RhoA regulator than an effector [12,13]. Moreover, Sti is not required for MRLC phosphorylation [12,23]. Together, these findings indicate that CIT-K is not required for the early stages of cleavage furrow formation and ingression, but rather for the proper organization and architecture of the midbody in late cytokinesis. Here we report the characterization of the CIT-K interactome in human cells and present evidence that this kinase plays a key role in organizing the proper distribution of midbody components. We also report that CIT-K binds to three CPC subunits, including its kinase component Aurora B, both *in vivo* and *in vitro*. Furthermore, we show that CIT-K is required for correct CPC localization and phosphorylates the INCENP subunit of the CPC at the TSS Aurora B activation site**.** Finally, Aurora B phosphorylates CIT-K to control its localization and interaction with central spindle partners. Together, our findings reveal a novel cross-regulatory mechanism between CIT-K and the CPC necessary to regulate midbody architecture.

## 2. Results

### 2.1. CIT-K interacts with the CPC *in vivo* and *in vitro*

**Figure 2.**
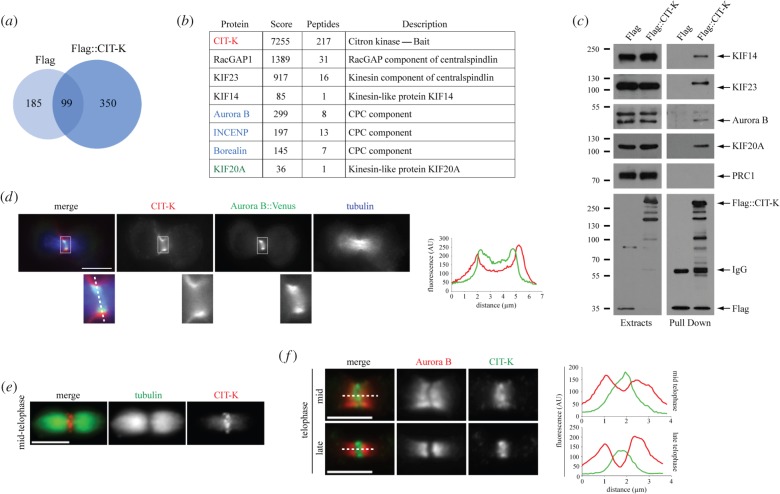
CIT-K associates with the CPC *in vivo*. (*a*) Proportional Venn diagram showing the overlap between the proteins pulled down by the Flag tag alone (284) and the proteins that co-purified with Flag::CIT-K (449). Note that 350 proteins specifically associated with Flag::CIT-K. (*b*) Partial list of the CIT-K specific interactors identified by affinity purification. A full list can be found in the electronic supplementary material, table S1, worksheet 1. The bait, CIT-K, is highlighted in red and previously known CIT-K binding partners are in black. The three subunits of the CPC are in blue and the kinesin responsible for the translocation of the CPC to the spindle midzone, KIF20A, is in green. (*c*) HeLa Kyoto cells stably expressing either Flag-tagged CIT-K or Flag alone were synchronized in telophase by thymidine/nocodazole block and release and then protein extracts were used in a pull-down assay using anti-Flag antibodies. The extracts and pull downs were analysed by western blot to detect KIF14, KIF23, Aurora B, KIF20A, PRC1 and Flag. Note that a slower migrating KIF23 band was detected in the pull-down compared with whole cell extracts. This is probably a phosphorylated form that we routinely observe in protein extracts from both telophase cells and purified midbodies. The numbers on the left indicate the sizes in kilodaltons of the molecular mass marker. (*d*) U2OS cells stably expressing Aurora B::Venus were washed in PBS to remove tetracycline (tet-off inducible system) and kept in tetracycline free media for 24 h. Cells were then fixed and stained to detect CIT-K (red), Aurora B::Venus (green) and tubulin (blue). Insets show a 3× magnification of the cleavage furrow region. The profiles of the green and red fluorescent signals, measured at the centre of the cleavage furrow (dotted line), are shown on the right. Scale bar, 10 µm. (*e*) Midbodies were purified form HeLa Kyoto cells and fixed and stained to detect CIT-K (red) and tubulin (green). Scale bar, 5 µm. (*f*) Midbodies were purified from HeLa Kyoto cells and fixed and stained to detect CIT-K (green) and Aurora B (red). The horizontal profiles of the green and red fluorescent signals, measured at the centre of the midbody (dotted lines), are shown on the right. Scale bars, 5 µm.

We previously showed that the midbody matrix appears scarce, fragmented and disorganized in *Drosophila* cells after CIT-K depletion [11]. We observed an almost identical phenotype in human HeLa cells after CIT-K depletion. Electron microscopy (EM) analysis revealed that the midbody matrix was fragmented, disorganized, detached from the cortex and mis-positioned towards one of the two nascent daughter cells (figure 1). Moreover, cortical blebs were often evident where gaps between the midbody matrix and the membrane occurred (figure 1, arrowhead). These findings confirmed a key role for CIT-K in midbody formation in human cells and prompted us to identify its interactome to understand the mechanisms that control midbody architecture. We generated a monoclonal HeLa cell line stably expressing a Flag-tagged CIT-K transgene that displayed correct localization pattern and rescued the cytokinesis failure caused by depletion of endogenous CIT-K, indicating that it is fully functional (electronic supplementary material, figure S1). By using affinity purification coupled with mass spectrometry (AP-MS), we identified 350 proteins that were specifically pulled down by Flag::CIT-K, but not Flag alone, in HeLa cells synchronized in telophase (figure 2*a*; electronic supplementary material, table S1). Among these proteins we found some expected previously identified partners, such as KIF14 [14] and the two components of the centralspindlin complex, KIF23 and RacGAP1 [11], and also other novel interactors, including three of the four CPC components, Aurora B, Borealin and INCENP (figure 2*b*; electronic supplementary material, table S1). It is very likely that the other CPC component, Survivin, was not identified simply because of MS limitations. The kinesin KIF20A/MKLP2, responsible for CPC translocation from centromeres to the spindle midzone [24], was also identified, albeit with a low score (figure 2*b*; electronic supplementary material, table S1). Notably, the microtubule-binding protein PRC1—a well-known direct binding partner of both KIF14 and KIF23 [11,14,25] but not of CIT-K [11]—was not found in our survey, suggesting that our analysis preferentially identified proteins strongly associated with CIT-K. These findings were confirmed by western blot (figure 2*c*) and suggested that an interaction between CIT-K and the CPC might be important for midbody formation.

CIT-K and the CPC both localize to the cleavage furrow [17,26], but unfortunately we failed to observe localization of the CPC to the equatorial cortex using antibodies against Aurora B and INCENP. However, we found that CIT-K co-localized with Venus-tagged Aurora B at the ingressing cortex in a transgenic U2OS cell line [27] (figure 2*d*), albeit fluorescence intensity profiles indicated that Aurora B-Venus was distributed closer to the inside edge of the furrow (figure 2*d*). CIT-K has been described to localize predominantly to the cleavage furrow (figure 2*d*) [17,19], but Flag::CIT-K also weakly accumulated at the spindle midzone (electronic supplementary material, figure S1, arrow). This does not seem to be an over-expression artefact because a similar midzone-associated localization of endogenous CIT-K has already been reported [28], and we could also observe it in isolated midbodies (figure 2*e* and *f*). This midzone signal partially overlapped with the CPC in mid-telophase, but in late-telophase CIT-K accumulated at the midbody ring, whereas the CPC localized to the midbody arms (figure 2*f*).

**Figure 3.**
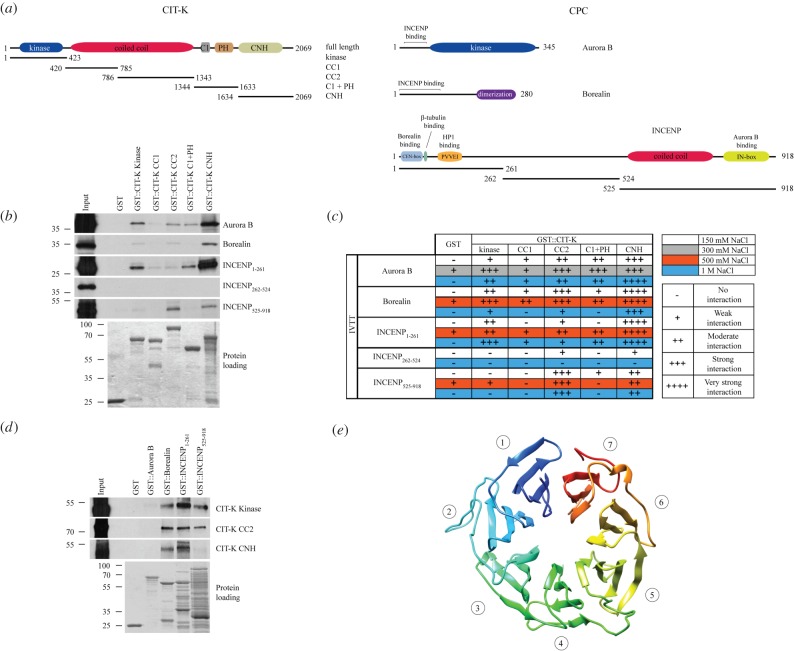
CIT-K directly binds to CPC components *in vitro*. (*a*) Schematic diagrams illustrating the protein domains of CIT-K and of the three CPC components analysed—Aurora B, Borealin and INCENP. The positions of the different CIT-K and INCENP fragments used for the *in vitro* pull-down assays are also indicated. Note that CIT-K is not drawn to the same scale as the CPC components. CC1 and CC2 indicate the fragments encompassing the first and second coiled coil regions; C1, cysteine-rich motif; PH, Pleckstrin homology domain; CNH, Citron-Nik1 homology domain. (*b*) The GST::CIT-K proteins indicated at the top and GST alone were incubated with the *in vitro* translated and radiolabelled Aurora B, Borealin and INCENP polypeptides indicated at the right, and then pulled down using glutathione beads. The Ponceau S staining of the protein loading is shown at the bottom and the numbers on the left indicate the sizes in kilodaltons of the molecular mass marker. (*c*) Summary of the results from the *in vitro* pull-down assays using GST-CIT-K fragments and *in vitro* transcribed and translated (IVTT) radiolabelled CPC polypeptides. The increasing salt concentrations used in the washes are colour-coded and indicated at the top right. The autoradiogram shown in (*b*) corresponds to the 1 M NaCl experiment highlighted in blue. (*d*) The GST-tagged CPC proteins indicated at the top and GST alone were incubated with the *in vitro* translated and radiolabelled CIT-K polypeptides indicated at the right, and then pulled down using glutathione beads. The protein loading is shown at the bottom and the numbers on the left indicate the sizes in kilodaltons of the molecular mass marker. (*e*) Ribbon diagram of the predicted structure of the CIT-K C-terminal CNH domain (residues 1634–1948), which adopts a seven-bladed β-propeller fold. Ribbon is coloured from blue (N-terminus) to red (C-terminus) and the numbers indicate the seven blades.

To assess whether CIT-K could directly bind to the CPC, we purified recombinant CIT-K fragments tagged with glutathione *S*-transferase (GST) from bacteria and tested their ability to pull down various *in vitro* translated and radiolabelled polypeptides of the three CPC subunits identified in our AP-MS experiments: Aurora B, Borealin and INCENP (figure 3*a–c*). All these fragments were chosen on the basis of the structures of the proteins and their known interaction domains. We carried out multiple experiments using increasing salt concentrations during the washes in order to have an indication of the strength of the interactions (figure 3*c*). The INCENP central region (residues 262–524) was never pulled down by any of the GST::CIT-K fragments (figure 3*b*,*c*). The CIT-K CNH domain interacted very strongly with Aurora B, Borealin and the N-terminal region of INCENP (residues 1–261) (figure 3*b*,*c*). A weaker interaction was observed between the CIT-K kinase domain and the same CPC polypeptides that bound to the CNH region (figure 3*b*,*c*). Finally, the CIT-K CC2 region pulled down very well the INCENP C-terminal region (residues 525–918) and less efficiently Aurora B, Borealin and INCENP_1-261_ (figure 3*b*,*c*). Reciprocal pull-down experiments failed to confirm any interaction for Aurora B, whereas both Borealin and INCENP_1–261_ could pull down the CIT-K kinase, CC2 and CNH regions (figure 3*d*). INCENP_525–918_ pulled down the CIT-K kinase and CC2 domains (figure 3*d*), but the interaction with the CIT-K kinase domain was never observed in the previous reciprocal assay (figure 3*b*,*c*). We conclude from all these *in vitro* binding experiments that the CIT-K CNH C-terminal region binds very strongly to the INCENP N-terminal region, Borealin and possibly Aurora B. The other two CIT-K regions, kinase and CC2, showed weaker interactions with the INCENP N-terminal and C-terminal regions, respectively. Moreover, they also weakly interacted with Borealin. However, it is difficult to imagine that all these interactions occur simultaneously and we speculate that, *in vivo*, the association between the CNH domain and the CPC components might be predominant. The exact function of the CIT-K CNH domain is unclear, but its predicted structure of a seven-bladed β-propeller (figure 3*e*), a structure known to function as a hub for multiple protein–protein interactions and found in scaffolding proteins of the WD40 family [29,30], is perfectly consistent with our binding results.

### 2.2. CIT-K is required for proper CPC localization and the orderly arrangement of midbody proteins

**Figure 4.**
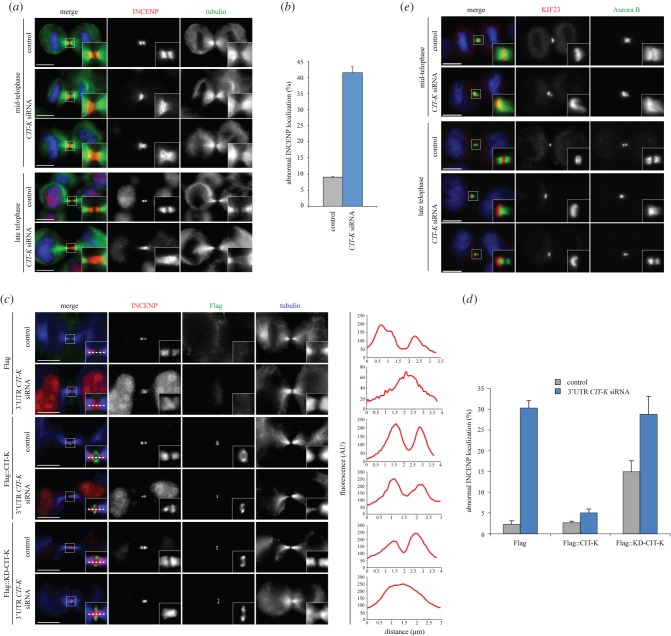
CIT-K is required for correct CPC localization and the orderly arrangement of midbody proteins. (*a*) HeLa Kyoto cells were treated with siRNAs directed against either a random sequence (control) or *CIT-K* and after 48 h were fixed and stained to detect DNA (blue), tubulin (green) and the CPC component INCENP (red). The shape and thickness of microtubule bundles at the intercellular bridge were used as criteria to stage telophase cells. Insets show a 3× magnification of the midbody. Scale bars, 10 µm. (*b*) Quantification of INCENP localization defects from the experiment shown in (*a*). More than 100 mid–late-telophase cells were counted in each experiment, *n* = 3. Scale bars indicate standard errors. (*c*) HeLa Kyoto cells stably expressing Flag-tagged wild-type CIT-K, a kinase-dead version (KD) of CIT-K, or Flag alone were treated with siRNAs directed against either a random sequence (control) or the 3′-UTR of *CIT-K* and after 48 h were fixed and stained to detect tubulin (blue), Flag (green) and the CPC component INCENP (red). The shape and thickness of microtubule bundles at the intercellular bridge were used as criteria to stage telophase cells. Insets show a 3× magnification of the midbody. The horizontal profiles of the red fluorescent signals, measured at the centre of the midbody (dotted lines), are shown at the right of the respective images. Scale bars, 10 µm. (*d*) Quantification of INCENP localization defects from the experiment shown in (*c*). Only Flag-positive cells were counted, and more than 100 mid–late-telophase cells were counted in each experiment, *n* = 3. Bars indicate standard errors. (*e*) HeLa Kyoto cells were treated with an siRNA directed against either a random sequence (control) or *CIT-K* and after 48 h were fixed and stained to detect DNA (blue), Aurora B (green) and KIF23 (red). The shape and thickness of microtubule bundles at the intercellular bridge were used as criteria to stage telophase cells. Insets show a 3× magnification of the midbody. Scale bars, 10 µm.

To investigate the functional significance of the interaction between CIT-K and the CPC, we first analysed CPC localization after CIT-K depletion. The CPC relocates from centromeres to the spindle midzone after anaphase onset, and then accumulates at the midbody arms [31]. The CPC's subunit INCENP localized normally during initial furrow ingression after CIT-K depletion (data not shown), but then failed to form two distinct bands in about 41% of mid- and late-telophase *CIT-K* RNAi cells (figure 4*a*,*b*). Often, INCENP appeared mis-positioned towards one of the two daughter cells (figure 4*a*), consistent with our EM analysis (figure 1). Identical phenotypes were observed for KIF20A (electronic supplementary material, figure S2), the kinesin that mediates CPC translocation to the spindle midzone [24]. Wild-type CIT-K, but not its kinase-dead version (KD-CIT-K), could rescue CPC localization after depletion of endogenous CIT-K (figure 4*c*,*d*), even though the two transgenic proteins showed identical localization patterns and were expressed at similar levels (figure 4*c*, electronic supplementary material, figure S1 and S3). Notably, expression of KD-CIT-K in control RNAi cells caused an increase of INCENP abnormal localization (figure 4*d*), most likely because of a dominant-negative effect. These results indicate that CIT-K kinase activity is necessary for proper CPC localization. To visualize not only CPC localization but also the entire midbody architecture, we simultaneously stained CIT-K depleted cells for Aurora B, which marks the midbody arms, and for the kinesin KIF23, a well-documented midbody ring/matrix marker (figure 4*e*). After CIT-K depletion, KIF23 no longer localized between the two CPC bands and appeared to be ‘pushed’ towards one side of the midbody in mid-telophase (figure 4*e*). In late-telophase *CIT-K* RNAi cells, KIF23 was often found to one side of the CPC (figure 4*e*), even when two Aurora B bands were clearly evident (figure 4*e*, bottom panels). These phenotypes were never observed in control cells. To conclude, our results indicate that CIT-K is required for proper CPC localization and for the orderly arrangement of midbody proteins.

### 2.3. CIT-K phosphorylates INCENP at the TSS Aurora B activation site

**Figure 5.**
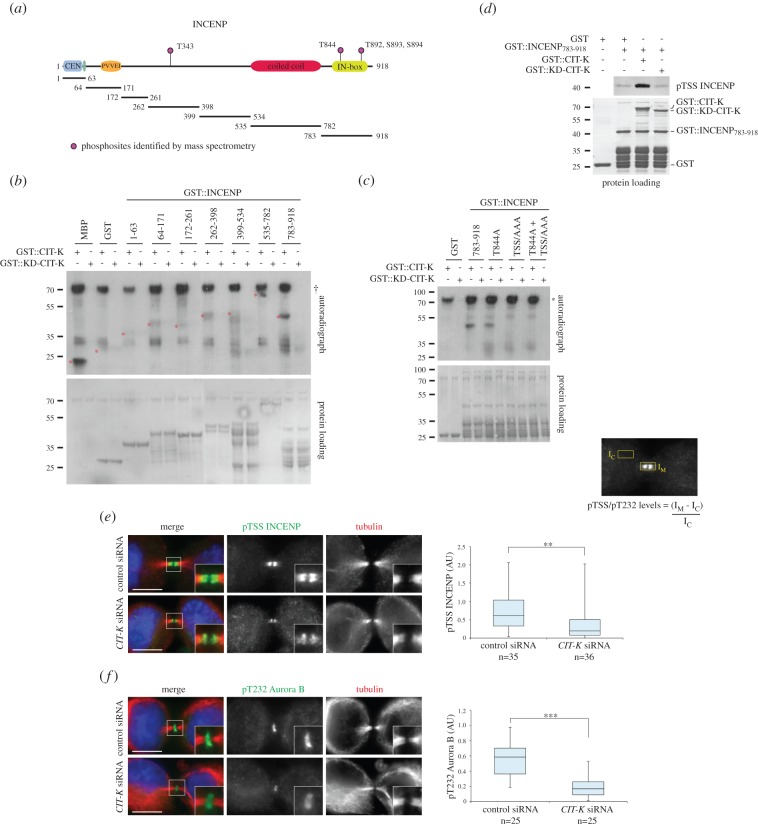
CIT-K phosphorylates INCENP. (*a*) Schematic diagram of INCENP structure illustrating the phosphorylated sites identified by MS. The GST-tagged fragments used for the *in vitro* phosphorylation assays shown in (*b*), (*c*) and (*d*) are depicted at the bottom. (*b*) GST-tagged INCENP polypeptides, GST alone, and the positive control MBP (myelin basic protein) were incubated with GST-tagged CIT-K kinase domain or KD-CIT-K kinase domain in the presence of [γ-^32^P] ATP. The reactions were then separated by SDS-PAGE, transferred onto nitrocellulose membranes, and exposed at −80°C. The Ponceau S staining of the protein loading is shown at the bottom. The asterisks mark the molecular positioning of the respective proteins. The dagger (†) indicates CIT-K auto-phosphorylation. The numbers on the left indicate the sizes in kilodaltons of the molecular mass marker. (*c*) GST alone, GST-tagged INCENP 783–918 and GST-tagged INCENP mutants (T844A, TSS/AAA and T844A + TSS/AAA), were incubated with GST-tagged CIT-K kinase domain or KD-CIT-K kinase domain in the presence of [γ-^32^P] ATP. The reactions were then separated by SDS-PAGE, transferred onto nitrocellulose membranes, and exposed at −80°C. The protein loading is shown at the bottom. An asterisk marks CIT-K auto-phosphorylation. The numbers on the left indicate the sizes in kilodaltons of the molecular mass marker. (*d*) GST alone and GST-tagged INCENP 783–918 were incubated in the presence or absence of GST-tagged CIT-K kinase domain or KD-CIT-K kinase domain, using non-radioactive ATP. The reactions were then separated by SDS-PAGE and analysed by western blot to detect phosphorylated INCENP. The protein loading is shown at the bottom. The numbers on the left indicate the sizes in kilodaltons of the molecular mass marker. (*e*) HeLa Kyoto cells were treated with siRNAs directed against either a random sequence (control) or CIT-K for 48 h. During RNAi incubation, cells were synchronized using 2 mM thymidine for 19 h, released for 5 h, treated with 10 µM RO3306 for 13 h, released for 2 h, fixed and stained to detect phosphorylated INCENP (green), tubulin (red) and DNA (blue). Insets show 2× magnification of the midbody. The box plot showing the quantification of pTSS fluorescence levels at the midbody is shown on the right. The intensity of pTSS INCENP fluorescence at the midbody was calculated using the formula shown, where the mean fluorescence intensity was measured at the midbody (*I*_M_) and the mean background fluorescence intensity was measured within an identical area inside the cytoplasm (*I*_C_). The numbers of cells counted are detailed below each plot. Scale bars, 10 µm. **p < 0.01 (Student's *t*-test). (*f*) HeLa Kyoto cells were treated as in (*e*), and stained to detect phosphorylated Aurora B (green), tubulin (red) and DNA (blue). Insets show 2× magnification of the midbody. The box plot showing the quantification of pT232 fluorescence levels at the midbody, calculated as described in (*e*), is shown on the right. The numbers of cells counted are detailed below each plot. Scale bars, 10 µm. ***p < 0.001 (Student's *t*-test).

To establish whether the components of the CPC could be CIT-K substrates, we developed an *in vitro* kinase assay using the isolated kinase domains (wild-type and kinase dead; KD) of human and mouse CIT-K purified from bacteria. These kinase domains were incubated with bacterially purified GST-tagged Aurora B, Borealin and INCENP. The CIT-K kinase domain phosphorylated INCENP N- and C-terminal fragments; however, no clear phosphorylation was observed for Aurora B and Borealin (electronic supplementary material, figure S4*a* and *b*). To pinpoint which regions of INCENP were phosphorylated by CIT-K, we repeated the *in vitro* kinase assay with smaller GST-tagged INCENP fragments (figure 5*a*). CIT-K strongly phosphorylated the INCENP_783–918_ C-terminal fragment, which contains the IN-box—responsible for Aurora B binding and activation [32]—and also weakly the INCENP_64–171_ and INCENP_262–398_ polypeptides (figure 5*b*). This phosphorylation pattern was confirmed using bacterially purified mouse CIT-K, suggesting this phosphorylation is evolutionarily conserved (electronic supplementary material, figure S4*c* and *d*). MS analysis identified four phosphorylated residues within the IN-box: T844 and the conserved TSS Aurora B activation site—T892, S893 and S894 (figure 5*a* and electronic supplementary material, S4*d*). Substituting the TSS motif to alanines (TSS/AAA) completely abolished INCENP_783–918_ phosphorylation by CIT-K, while there was only a slight decrease in phosphorylation for the T844A mutant (figure 5*c* and electronic supplementary material, figure S4*e*). We verified that CIT-K phosphorylated INCENP *in vitro* by using an antibody that recognizes the phosphorylated TSS motif (pTSS) of human INCENP [33] (figure 5*d*). CIT-K depletion in HeLa cells caused a 35% reduction in the pTSS signal by immunofluorescence (figure 5*e*). As phosphorylation of the TSS motif increases Aurora B kinase activity [34–36], our data suggested that CIT-K could be important to elicit full Aurora B activation at the midbody. Consistent with this hypothesis, CIT-K depletion caused a significant decrease in Aurora B phosphorylation at T232 (figure 5*f*), which is essential to achieve full Aurora B activation following INCENP TSS phosphorylation [35,37]. Notably, although CPC components often failed to properly localize to the midbody after CIT-K RNAi, their accumulation was not reduced (figure 4). Therefore, we conclude that CIT-K is required for proper localization and phosphorylation of INCENP and Aurora B, but not their recruitment, at the midbody.

### 2.4. CIT-K localization during cytokinesis requires Aurora B activity

**Figure 6.**
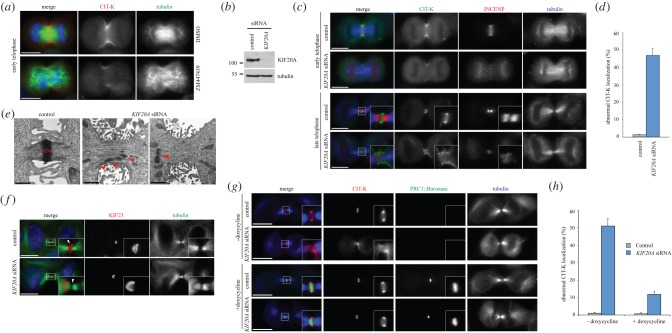
Aurora B activity is necessary for proper CIT-K localization. (*a*) Asynchronous HeLa Kyoto cells were treated for 20 min with the Aurora B inhibitor ZM447439 at a final concentration of 5 µM and then fixed and stained to detect CIT-K (red), tubulin (green) and DNA (blue). Scale bars, 10 µm. (*b*) HeLa Kyoto cells were treated with siRNAs directed against either a random sequence (control) or *KIF20A* for 48 h and then proteins were extracted, separated by SDS-PAGE, transferred to a membrane, and incubated to detect KIF20A and tubulin (loading control). (*c*) HeLa Kyoto cells were treated with siRNAs directed against either a random sequence (control) or *KIF20A* and after 48 h were fixed and stained to detect CIT-K (green), INCENP (red) and tubulin (blue). The shape and thickness of microtubule bundles at the intercellular bridge were used as criteria to stage telophase cells. Insets show a 3× magnification of the midbody. Scale bars, 10 µm. (*d*) Quantification of CIT-K localization defects from the experiment shown in (*c*). More than 100 late-telophase cells were counted in each experiment, *n* = 3. Bars indicate standard errors. (*e*) EM images of midbodies from HeLa Kyoto cells treated with an siRNA directed against either a random sequence (control) or *KIF20A* for 48 h. The arrowheads mark the midbody matrix (MM) in KIF20A depleted cells. Scale bars, 1 µm. (*f*) HeLa Kyoto cells were treated with an siRNA directed against either a random sequence (control) or *KIF20A* and after 48 h were fixed and stained to detect DNA (blue), tubulin (green) and KIF23 (red). The arrowheads mark the dark zone. Insets show a 3× magnification of the midbody. Scale bars, 10 µm. (*g*) HeLa Kyoto cells carrying a doxycycline-inducible GFP-tagged PRC1-Baronase transgene were treated with siRNAs directed against either a random sequence (control) or *KIF20A* and after 24 h incubated in 2 mM thymidine for a further 20 h. Cells were washed and incubated with or without 1 µg/ml doxycycline for 10 h and then fixed and stained to detect CIT-K (red), PRC1-Baronase (green) and tubulin (blue). Scale bars, 10 µm. (*h*) Quantification of CIT-K localization defects from the experiments shown in (*f*). More than 100 late-telophase cells were counted in each experiment, *n* = 3. Bars indicate standard errors.

We next analysed CIT-K localization after perturbation of CPC activity. After treatment with the Aurora B inhibitor ZM447439, CIT-K showed reduced accumulation at the ingressing furrow in early telophase (figure 6*a*). Treatment with ZM447439 impairs the spindle assembly checkpoint and cells exit mitosis very rapidly, often failing to assemble a proper central spindle and midbody (figure 6*a*). Thus, to investigate the role of the CPC in CIT-K localization in late cytokinesis, we prevented the translocation of this complex to the spindle midzone by depleting the kinesin KIF20A [24]. As expected, INCENP failed to accumulate at the spindle midzone and persisted at centromeres after KIF20A depletion (figure 6*b*,*c*). Consistent with the results obtained after Aurora B inhibition (figure 6*a*), CIT-K showed reduced accumulation at the cleavage furrow in early telophase *KIF20A* RNAi cells (figure 6*c*). Moreover, CIT-K failed to make a compact structure at the midbody ring and appeared diffuse along the cortex in about 47% of late-telophase *KIF20A* RNAi cells, a phenotype very rarely observed (1.3%) in control cells (figure 6*c*,*d*). EM ultrastructural analysis confirmed that, after KIF20A depletion, the midbody matrix was scarce, fragmented and mis-localized, similar to and possibly even slightly more severe than in *CIT-K* RNAi cells (figure 6*e* cf. figure 1). Moreover, KIF20A depleted cells also showed KIF23 mis-localization towards one side of the midbody, exactly like after *CIT-K* RNAi (figure 6f cf. figure 4*e*). These results indicate that CPC recruitment to the midbody is necessary for proper CIT-K localization and that the CPC and CIT-K cooperate to organize the architecture of the midbody.

To distinguish whether CIT-K localization required the whole CPC or just Aurora B activity, we tested if a chimera composed of the microtubule-binding domain of the spindle midzone protein PRC1 coupled with an Aurora B kinase module (dubbed Baronase and comprising a truncated form of Aurora B and the INCENP activating region) [38] could rescue CIT-K localization after KIF20A depletion. Cells carrying a doxycycline-inducible PRC1::Baronase transgenic chimera were depleted of KIF20A and CIT-K localization was analysed with or without expression of the PRC1::Baronase chimera. In the absence of transgene expression, cells showed a frequency of CIT-K abnormal localization very similar to non-transgenic HeLa cells (figure 6*g*,*h*). Induction of PRC1::Baronase expression, however, considerably rescued CIT-K localization (figure 6*g*,*h*). These results indicate that CIT-K localization requires primarily Aurora B activity.

### 2.5. Aurora B phosphorylates CIT-K to regulate its interaction with central spindle components

**Figure 7.**
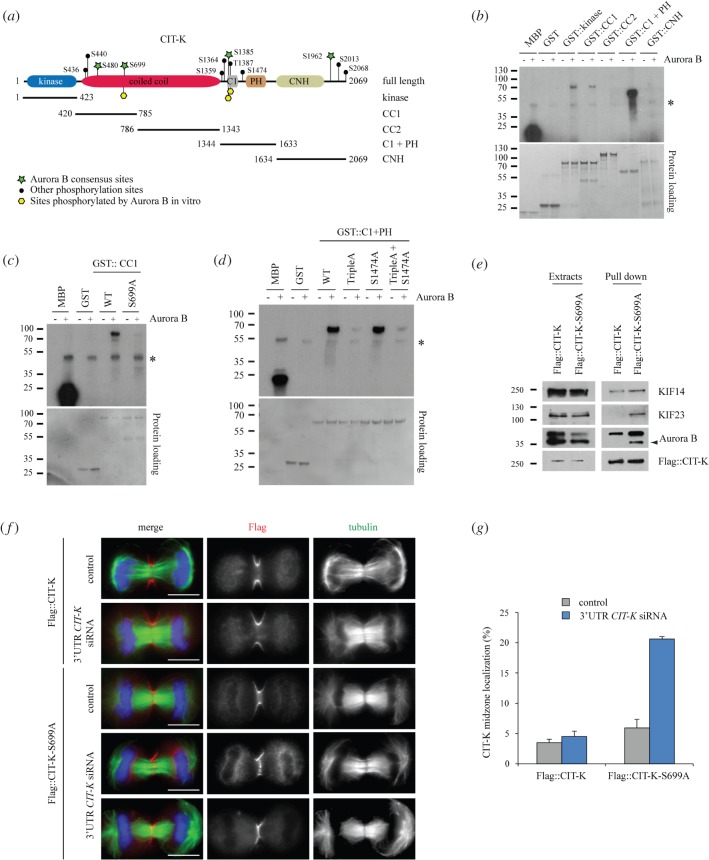
Aurora B phosphorylates CIT-K. (*a*) Schematic diagram of CIT-K structure illustrating the phosphorylated sites identified by MS. The GST- tagged fragments used for the *in vitro* phosphorylation assays shown in (*b*), (*c*) and (*d*) are depicted at the bottom. (*b*) GST-tagged CIT-K polypeptides, GST alone and the positive control MBP were incubated with (+) or without (−) recombinant Aurora B in the presence of [γ-^32^P] ATP. The reactions were then separated by SDS-PAGE, transferred onto nitrocellulose membranes and exposed at −80°C. The Ponceau S staining of the protein loading is shown at the bottom. Aurora B auto-phosphorylation is marked by an asterisk. The numbers on the left indicate the sizes in kilodaltons of the molecular mass marker. (*c*) GST-tagged wild-type CIT-K-CC1 (WT) and S699A mutant polypeptides, GST alone and the positive control MBP (myelin basic protein) were incubated with (+) or without (−) recombinant Aurora B in the presence of [γ-^32^P] ATP. The reactions were then separated by SDS-PAGE, transferred onto nitrocellulose membranes and exposed at −80°C. The protein loading is shown at the bottom. Aurora B auto-phosphorylation is marked by an asterisk. The numbers on the left indicate the sizes in kilodaltons of the molecular mass marker. (*d*) The GST-tagged wild-type CIT-K-C1+PH peptide (WT), along with the S1385A-S1386A-T1387A (TripleA) and S1474A mutant polypeptides, GST alone and the positive control MBP (myelin basic protein) were incubated with (+) or without (−) recombinant Aurora B in the presence of [γ-^32^P] ATP. The reactions were then separated by SDS-PAGE, transferred onto nitrocellulose membranes and exposed at −80°C. The protein loading is shown at the bottom. Aurora B auto-phosphorylation is marked by an asterisk. The numbers on the left indicate the sizes in kilodaltons of the molecular mass marker. (*e*) HeLa Kyoto cells stably expressing Flag::CIT-K or Flag::CIT-K-S699A were treated with an siRNA directed against the CIT-K 3′-UTR for 48 h, blocked in metaphase by thymidine/nocodazole block, released for 90 min and then treated with 10 µM RO3306 for further 15 min. Proteins were extracted and used in a pull-down assay with anti-Flag antibodies. The extracts and pull downs were analysed by western blot to detect KIF14, KIF23, Aurora B and Flag::CIT-K. The numbers on the left indicate the sizes in kilodaltons of the molecular mass marker. (*f*) HeLa Kyoto cells stably expressing Flag::CIT-K or Flag::CIT-K-S699A were treated with siRNAs directed against either a random sequence (control) or 3′-UTR CIT-K for 48 h. During RNAi incubation, cells were synchronized using 2 mM thymidine for 19 h, released for 5 h, treated with 10 µM RO3306 for 13 h, released for 2 h, fixed and stained to detect Flag (red), tubulin (green) and DNA (blue). All images are maximum intensity projections of the three most central *z* sections; *z* step = 0.25 µm. Scale bars, 10 µm. (*g*) Quantification of CIT-K midzone localization from the experiments showed in (*f*). No less than 50 early–mid-telophase cells were counted in each experiment, *n* = 4. Bars indicate standard errors.

As the results described in the previous paragraph indicated that CIT-K localization depends on Aurora B activity, we next determined whether Aurora B phosphorylated CIT-K. We first used MS to analyse the phosphorylation pattern of our Flag::CIT-K transgene purified from cells synchronized in telophase. Four out of the twelve identified phosphorylated sites matched the consensus for Aurora B kinase: S480, S699, S1385 and S1962 (figure 7*a*; electronic supplementary material, table S2). To address if CIT-K could be a direct substrate of Aurora B, we carried out *in vitro* phosphorylation assays using recombinant Aurora B and bacterially purified GST::CIT-K peptides. Aurora B phosphorylated three CIT-K regions containing the kinase, CC1 and C1+PH regions (figure 7*b*). Consistent with these *in vitro* results, two residues found to be phosphorylated *in vivo*, S480 and S699, lie within the CC1 region and one, S1385, lies within the C1 domain (figure 7*a*). MS analysis confirmed that S699, but not S480, was phosphorylated by Aurora B *in vitro* and, consistent with this, a CC1 fragment containing an S to A mutation at this residue (S699A) was no longer efficiently phosphorylated by Aurora B *in vitro* (figure 7*c*). MS analysis also identified residues in the C1 + PH region phosphorylated by Aurora B *in vitro*. The most highly phosphorylated residues were a stretch of two S and one T residue at positions 1385–1387 and S1474, which were also found phosphorylated *in vivo* (figure 7*a*). However, only substitution of the residues at positions 1385–1387 into alanines (TripleA mutant) significantly reduced phosphorylation by Aurora B *in vitro* (figure 7*d*). Together these results indicate that at least two CIT-K regions are phosphorylated by Aurora B *in vitro* and *in vivo*: S699 and the three SST residues at 1385–1387. It is, however, possible that other post-translational modifications and/or factors might be necessary for Aurora B to recognize and phosphorylate other CIT-K residues, such as S480 and S1474, *in vivo*.

As CIT-K interacts with both KIF14 and KIF23 via its CC1 domain [11,15], our results suggested that Aurora B could regulate the interaction of CIT-K with these two kinesins and possibly other partners. To address this hypothesis, we generated a monoclonal cell line expressing a Flag-tagged CIT-K S699A non-phosphorylatable mutant to test its localization and interaction with central spindle partners after depletion of endogenous CIT-K. The S699A mutation enhanced the association of CIT-K with KIF23 and Aurora B, but only marginally affected the interaction with KIF14 (figure 7*e*). Longer exposures confirmed that Flag-tagged wild-type CIT-K was also able to bind KIF23 and Aurora B (electronic supplementary material, figure S5), as already shown (figure 2*c*). Consistent with this result, the CIT-K S699A mutant showed strongly increased accumulation at the spindle midzone and irregular cortical localization in early stages of cytokinesis (figure 7*f*,*g*). Together these findings indicate that CIT-K is an Aurora B substrate and that Aurora B phosphorylation at S699 dampens the association of CIT-K with KIF23 and the CPC in order to reduce CIT-K accumulation at the spindle midzone in early cytokinesis.

## 3. Discussion

**Figure 8.**
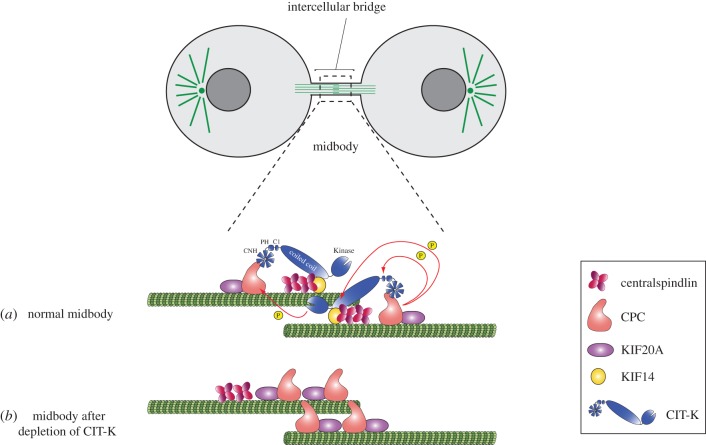
Cartoon illustrating the cross-regulation between Aurora B and CIT-K in midbody assembly. The red arrows indicate the phosphorylation of the CIT-K CC1 and C1 domains by Aurora B and the phosphorylation of the INCENP TSS motif by CIT-K. In the presence of CIT-K (*a*), midbody components including the CPC, centralspindlin, KIF20A and KIF14 are properly aligned along the midbody. By contrast, after depletion of CIT-K (*b*) KIF14 is not recruited to the midbody [12,14], the CPC and KIF20A are dispersed and centralspindlin is mis-positioned on one side of the midbody.

Cross-regulation among mitotic kinases, and in particular between Aurora B and Polo-like kinase 1 (Plk1), is emerging as an important mechanism to fine-tune important events and to improve fidelity and robustness during cell division [39–41]. Here we show for the first time that cross-regulation between two kinases, Aurora B and CIT-K, is crucial for regulation of midbody formation. Our findings identify CIT-K as a key factor that controls the orderly arrangement of proteins, including the CPC, at the midbody. These results reinforce and expand the evidence that CIT-K plays a unique role in shaping midbody architecture. Our data support a model in which, thanks to its ability to bind simultaneously to proteins localized to the midbody ring (i.e. KIF23 and KIF14) and to the midbody arms (i.e. the CPC), CIT-K could act as both bridge and organizer (figure 8). However, it is important to point out that KIF23 and the CPC, albeit improperly localized, remained separate in the absence of CIT-K (figure 4*e*). This indicates that additional mechanisms must exist to prevent the intermingling of midbody ring and midbody proteins. We cannot categorically exclude that aberrant localization of midbody proteins could be an indirect effect of the abnormal midbody structure observed after CIT-K depletion. However, the evidence that CIT-K directly associates with these midbody components (this paper and [11]) and that KIF23 fails to localize to the midbody ‘dark region’ even when the CPC appears to localize normally (figure 4*e*), strongly argue against this possibility.

CIT-K and the CPC display a complex binding pattern *in vitro* (figure 3). The very strong interaction observed between the CIT-K CNH region and all the three CPC subunits analysed (figure 3*b*,*c*) strongly suggests that this binding might be prevalent *in vivo*, and it is tempting to speculate that different ‘blades’ of the CNH propeller could interact with distinct components of the CPC. It is noteworthy that, consistent with these findings, our previous study in *Drosophila* indicated that a CIT-K mutant lacking the CNH domain could not fully rescue cytokinesis failure after depletion of the endogenous kinase [12]. We cannot exclude, however, that additional minor interactions between other CIT-K domains and the CPC could also occur, and *in vivo* cross-linking experiments and detailed structural analyses will be required to define this complex interaction.

Our results indicate that Aurora B phosphorylation controls CIT-K localization and its interaction with spindle midzone partners through phosphorylation of the CC1 region (figures 4, 6 and 7). This also implies that the interaction of CIT-K with the CPC is probably not important for CIT-K localization. Our data indicate that Aurora B phosphorylation prevents accumulation of CIT-K at the spindle midzone in early cytokinesis. We speculate that this may act as a timing mechanism to ensure that CIT-K associates strongly with KIF23 and the CPC only in late telophase, when CPC activity diminishes. The localization of CIT-K S669A does not entirely reflect the distribution of CIT-K after Aurora B inactivation or CPC removal from the spindle midzone (figure 6). This indicates that Aurora B might control CIT-K localization also via phosphorylation of other CIT-K regions and/or other cytokinesis proteins. Other CIT-K residues phosphorylated *in vivo* that match the Aurora B consensus site were found downstream of the CNH domain and within the C1 region (figure 7*a*). However, the CNH region was not phosphorylated by Aurora B *in vitro* (figure 7*b*), indicating either that another kinase is responsible for this phosphorylation, or that Aurora B might require other post-translational modifications in order to recognize this site. By contrast, the C1 region was strongly phosphorylated *in vivo* and by Aurora B *in vitro* (figure 7), suggesting that the function of this domain is tightly controlled by Aurora B and possibly other kinases. The role of CIT-K's C1 domain has not been characterized yet, but it most likely mediates the interaction of CIT-K with other proteins and/or membrane phospholipids like the C1 domain of RacGAP1 [42]. Although the roles of these phosphorylation events require further investigation, our results strongly indicate that Aurora B could regulate multiple CIT-K functions. It is also worth mentioning that most of the other CIT-K phosphorylated sites identified *in vivo* match the consensus of other important mitotic kinases, such as Plk1 and Cyclin-dependent kinases, raising the possibility that multiple signalling pathways might converge to regulate the function of this key regulator of midbody architecture.

CIT-K kinase activity is necessary for proper CPC localization and to elicit full activation of Aurora B at the midbody through phosphorylation of the INCENP TSS motif (figures 4 and 5). As Aurora B phosphorylation in turn controls CIT-K distribution (figure 7), this data suggests the existence of a feedback regulatory loop by which CIT-K reinforces both its own localization and proper architecture of the entire midbody (figure 8). Our findings are consistent with previous results that CIT-K kinase activity is necessary for successful cytokinesis in *Drosophila* and human cells [12,15]. INCENP is the first CIT-K substrate that has been confirmed *in vivo*. Previous reports indicating that human CIT-K phosphorylated the MRLC [18] were based solely on gain-of-function experiments and have been challenged by the evidence that CIT-K depletion did not affect MRLC phosphorylation in *Drosophila* cells [12,23]. It will be important in the future to identify the substrates of this important cytokinetic kinase to fully understand its functions during cytokinesis.

## 4. Material and methods

### 4.1. Molecular biology

The cDNA clones for Aurora B, INCENP and Borealin were kind gifts of Dr C. Lindon (Department of Genetics, University of Cambridge, UK). Cloning of the full-length open reading frame (ORF) of human CIT-K was performed by sequentially inserting three different fragments of the CIT-K cDNA sequence into the I.M.A.G.E. cDNA clone 4149886 (for simplicity, renamed construct A) (Source Bioscience). This construct encodes the 5′-terminal region (nt 4–811) of the CIT-K transcript (RefSeq accession number: NM_001206999.1) cloned within the pCMV-SPORT6 vector. First, poly(A) messenger RNA (mRNA) was extracted from HeLa Kyoto cells using the MicroPoly(A)Purist Kit (Ambion) and transcribed into cDNAs using random primers and the SuperScript First-Strand Synthesis System for RT-PCR (Invitrogen). The resulting cDNA library was then used as a template to PCR-amplify two CIT-K fragments dubbed CIT-K kin (nt 267–1385) and CIT-K mid (nt 1162–4923), using the primers listed in the electronic supplementary material, table S1 and the Platinum Taq DNA Polymerase High Fidelity (Invitrogen). These PCR products were subsequently cloned individually into the pGEM-T Easy vector using the pGEM-T Easy Vector System (Promega). Through digestion with the BamHI and NotI restriction enzymes, the CIT-K kin fragment was excised from the pGEM-T Easy vector and cloned into construct A in frame with the upstream 5′-terminal CIT-K portion. The CIT-K mid region was then inserted into the resulting plasmid using the BglII and NotI restriction sites. Finally, the CIT-K C-terminal region (CIT-K Ct), spanning nt 4457–6293, was PCR-amplified from the I.M.A.G.E. cDNA clone 40133624 (Source Bioscience), digested with BstEII and NotI, and ligated to the rest of the sequence to obtain the full-length human CIT-K ORF. CIT-K kinase dead (K126A) [43] and S699A mutants were generated by site-directed mutagenesis using the QuikChange Lightning Site-Directed Mutagenesis Kit (Agilent) following manufacturer's instructions.

For expression of Flag-tagged CIT-K proteins in HeLa Kyoto cells, a Gateway destination vector was generated as follows: the DNA sequence coding for three copies of the Flag epitope and four copies of the Myc epitope (indicated in the text simply as Flag), followed by the Gateway cassette for N-terminal fusion, was PCR-amplified from the pMYFN Gateway destination vector and then cloned into the pIRESpuro3 vector (Clontech) using the NheI and EcoRV restriction enzymes.

For bacterial expression of GST-tagged proteins, the pDEST15 Gateway destination vector (Invitrogen) was used. All DNA constructs were verified by sequencing.

### 4.2. Cell culture, siRNA transfection, drug treatments and generation of stable cell lines

HeLa Kyoto and cells expressing the PRC1::Baronase chimera were maintained in DMEM (Invitrogen) containing 10% fetal bovine serum (Sigma) and 1% penicillin/streptomycin (Invitrogen) at 37°C and 5% CO_2_. To express the PRC1::Baronase construct, cells were treated with 1 µg ml^−1^ doxycycline for 10 h. U2OS Aurora B::Venus cells were kept under the same conditions as HeLa Kyoto, with the addition of 1 µg ml^−1^ tetracycline to inhibit expression of Aurora B::Venus (tet-off inducible system). For RNA interference the following siRNAs were used: scrambled sequence control, 5′-AACGTACGCGGAATACTTCGA-3′; CIT-K, 5′-ATGGAAGGCACTATTTCTCAA-3′; CIT-K 3′-UTR, 5′-CACACUAUGGAACUCUGCU-3′; KIF20A, 5′-AACCACCTATGTAATCTCATG-3′, using Lipofectamine RNAiMAX (Invitrogen) following the manufacturer's instructions.

Cell lines stably expressing Flag alone, Flag::CIT-K, Flag::KD-CIT-K or Flag::CIT-K-S699A constructs were generated by plating 2 × 10^6^ HeLa Kyoto cells in a 100 mm culture dish and transfected with 19 µg of respective DNA using FuGENE HD transfection reagent (Promega) for 48 h. Cells were subsequently washed with PBS and cultured in complete selective medium containing 0.4 µg ml^−1^ puromycin for approximately two weeks until colonies became visible. Individual colonies were picked, cultured under resistance and tested for expression of the construct by western blot and immunofluorescence.

### 4.3. Affinity purification

For large-scale affinity purifications of Flag-tagged CIT-K, 4 × 10^7^ cells were synchronized in telophase using a thymidine–nocodazole block and release procedure. They were first arrested in S phase by the addition of 2 mM thymidine (Sigma-Aldrich) for 19 h, washed twice with phosphate-buffered saline (PBS) and released for 5 h in fresh complete medium. After release, cells were cultured for an additional 13 h in fresh complete medium containing 50 ng ml^−1^ nocodazole (Sigma-Aldrich) and then harvested by mitotic shake-off. Mitotic cells were washed three times with PBS, released in fresh medium for 1.5 h and incubated for a further 15 min with the CDK1 inhibitor RO3306 (Calbiochem) at a final concentration of 10 µM. Cells were then harvested by centrifugation and frozen in liquid nitrogen. The cell pellet was resuspended in 5 ml of extraction buffer (50 mM HEPES pH 7.5, 100 mM KAc, 150 mM NaCl, 2 mM MgCl_2_, 1 mM EGTA, 0.5% (v/v) NP-40, 1 mM DTT, 5% (v/v) glycerol and Roche Complete Protease Inhibitors) and homogenized using a high-performance disperser (Fisher). The homogenate was clarified by centrifugation at 750*g* for 20 min at 4°C and the supernatant was incubated with 200 µl of anti-FLAG M2 Magnetic Beads (Sigma-Aldrich) for 2–4 h on a rotating wheel at 4°C. Beads were then washed four times in 10 ml of extraction buffer for 5 min on a rotating wheel, transferred to a new tube and washed one more time in 10 ml of PBS. Proteins were eluted from beads with 0.5 M NH_4_OH and 0.5 mM EDTA, concentrated, acetone precipitated and analysed by LC-MS/MS.

A very similar procedure was used for western blot analyses. Ten million cells were resuspended in 3 ml of extraction buffer and 50 µl of anti-FLAG M2 Magnetic Beads were added to the supernatant. Washes were performed with 10 ml of extraction buffer. After the last wash in PBS, beads were resuspended in 2× Laemmli sample buffer (Sigma-Aldrich), boiled for 10 min and stored at −20°C. Proteins were separated on an SDS-PAGE gel, transferred onto PVDF membrane and probed to detect the antigens indicated in the figure legends.

### 4.4. Midbody purification

HeLa S3 cells were synchronized in metaphase by thymidine–nocodazole block as described in the previous section, harvested by mitotic shake-off and incubated for an additional 2 h in fresh medium supplemented with 10 µM MG132 (Sigma). Cells were then washed three times with PBS, released in normal medium and allowed to progress throughout mitosis. After approximately 1.5 h, when the vast majority of cells had completed furrowing, 5 µg ml^−1^ taxol was added to the medium for 2–3 min to stabilize microtubules *in vivo*. Cells were then harvested by centrifugation, washed once with warm H_2_O and gently resuspended in a hypotonic swelling solution containing 1 mM PIPES pH 7, 1 mM MgCl_2_, 5 µg ml^−1^ taxol (Sigma) and Roche Complete Protease Inhibitors. Cells were immediately centrifuged at 200*g* for 3 min, resuspended in lysis buffer (1 mM PIPES pH 7, 1 mM EGTA, 1% (v/v) NP-40, 5 mg ml^−1^ taxol and Roche Complete Protease Inhibitors) and vortexed vigorously. After the addition of 0.3 volumes of cold 50 mM 2-(*N*-morpholino) ethane sulfonic acid (MES) pH 6.3, cells were incubated on ice for 20 min and then centrifuged at 200*g* for 10 min at 4°C. The supernatant was transferred to a new tube and centrifuged at 650*g* for 20 min at 4°C to pellet midbodies. The pellet was resuspended in 50 mM MES pH 6.3 and centrifuged through a cushion of 40% (w/v) glycerol in 50 mM MES pH 6.3 at 2800*g* for 45 min at 4°C. After a final wash in 50 mM MES pH 6.3, midbodies were plated on poly-lysine-coated coverslips and processed for immunofluorescence as described below.

### 4.5. Antibodies

The following antibodies were used in this study: mouse monoclonal anti α-tubulin (clone DM1A, Sigma, T9026), chicken polyclonal anti-α-tubulin (Abcam, ab89984), mouse monoclonal anti-Flag (clone M2, Sigma, F3165), mouse monoclonal anti-CIT-K (BD Transduction Laboratories, 611377), rabbit polyclonal anti-Aurora B (Abcam, ab2254), rabbit polyclonal anti-pT232 Aurora B (Abcam, ab61074), rabbit polyclonal anti-INCENP (clone P240, Cell Signaling, 2807), rabbit polyclonal anti-pTSS INCENP (a kind gift of M.A. Lampson) [33], rabbit polyclonal anti-KIF23 (clone N19, Santa Cruz Biotechnology, sc-867), rabbit polyclonal anti-KIF14 (Bethyl Laboratories, A300–233A) and rabbit polyclonal anti-KIF20A (a kind gift of T.U. Mayer) [44]. Peroxidase and Alexa-fluor conjugated secondary antibodies were purchased from Jackson Laboratories and Invitrogen.

### 4.6. Fluorescence microscopy

HeLa Kyoto cells were grown on microscope glass coverslips (Menzel-Gläser) and fixed in PHEM buffer (60 mM Pipes, 25 mM HEPES pH 7, 10 mM EGTA, 4 mM MgCl_2_, 3.7% (v/v) formaldehyde) for 12 min. They were then washed three times for 10 min with PBS and incubated in blocking buffer (PBS, 0.5% (v/v) Triton X-100 and 1% (w/v) BSA) for 1 h at room temperature (RT). Coverslips were incubated overnight at 4°C with the primary antibodies indicated in the figure legends, diluted in PBT (PBS, 0.1% (v/v) Triton X-100 and 1% (w/v) BSA). The day after, coverslips were washed twice for 5 min in PBT, incubated with secondary antibodies diluted in PBT for 2 h at RT and then washed twice with PBT and once with PBS. Coverslips were mounted on SuperFrost Microscope Slides (VWR) using VECTASHIELD Mounting Medium containing DAPI (Vector Laboratories). Phenotypes were scored blind and by at least two people independently. ImageJ software was used to generate RGB fluorescence profiles and intensity values.

### 4.7. Transmission electron microscopy

For electron microscopy analyses, asynchronous HeLa Kyoto cells were plated on microscope glass coverslips (Menzel-Gläser), incubated for 19 h with 2 mM thymidine, washed three times with PBS, released for 5 h in fresh complete medium, and incubated for 13 h with 10 µM RO3306. Mitotic cells were then gently washed with warm PBS, and released in fresh medium for 2 h. Cells were fixed overnight at 4°C in 2.5% (v/v) glutaraldehyde in PBS, post-fixed for 1 h in 1% (v/v) OsO_4_ in PBS, dehydrated in a graded series of alcohols, embedded in Epon-Araldite resin, and polymerized for 2 days at 60°C. Glass slides were separated from the resin after a short immersion in liquid nitrogen. Sections were obtained with an LKB ultratome, stained with uranyl acetate and lead citrate, and observed and photographed with an FEI Tecnai G2 Spirit transmission electron microscope operating at an accelerating voltage of 100 kV and equipped with a Morada CCD camera (Olympus).

### 4.8. *In vitro* binding assay

DNA fragments coding for CIT-K fragments (kinase, CC1, CC2, C1 + PH and CNH), Aurora B, Borealin, INCENP_1-261_ and INCENP_525-918_ were generated by PCR and cloned into pDEST15 (Invitrogen) to express N-terminal GST-tagged polypeptides in *Escherichia coli*. The GST-tagged products were then purified using Glutathione Sepharose 4B according to manufacturer's instruction (GE Healthcare). [^35^S] Methionine-labelled Aurora B, Borealin, INCENP (all three fragments) and CIT-K fragments (kinase, CC2 and CNH) were prepared from corresponding PCR products amplified using primers harbouring a T7 promoter and then *in vitro* transcribed and translated (IVTT) using the TnT T7 Quick Coupled Transcription/Translation System (Promega) in the presence of [^35^S] methionine (PerkinElmer). The binding reaction contained 150 mM NaCl and subsequent washes varied from 150 mM to 1 M NaCl. GST pull-down assays were carried out as described [12].

### 4.9. *In vitro* phosphorylation assay

GST-tagged CIT-K fragments were incubated with 190 ng of recombinant human Aurora B (Invitrogen), 0.1 mM ATP (Sigma-Aldrich), 5 µCi of [*γ*-^32^P] ATP (6000 Ci mmol^−1^, 10 mCi ml^−1^) (PerkinElmer) and kinase buffer (20 mM HEPES pH 7.5, 2 mM MgCl_2_, 1 mM DTT) in a final reaction volume of 15 µl. After 30 min incubation at 30°C with constant agitation, 15 µl of 2× Laemmli sample buffer was added to stop the reaction. Samples were boiled for 10 min and loaded on a 4–20% Tris–Glycine precast gel (Thermo Scientific). Gels were stained with Quick Coomassie Stain (Generon) to check the protein loading and then proteins were transferred onto a nitrocellulose membrane using the iBlot Dry Blotting System (Invitrogen). Membranes were exposed to Kodak BioMax XAR Films (Sigma-Aldrich) at −80°C. The radioactive CIT-K *in vitro* kinase assay was performed as described above except that GST-tagged CIT-K and KD-CIT-K were incubated with GST-tagged Aurora B, Borealin and INCENP in a final volume of 25 µl for 1 h at 30°C with constant agitation, where 25 µl of 2× Laemmli sample buffer was added to stop the reaction. The non-radioactive CIT-K *in vitro* kinase assay was performed as above except using GST-tagged INCENP 783–918 as the substrate, with ATP at a final concentration of 0.5 mM.

### 4.10. Mass spectrometry

For the identification of CIT-K interactors, the raw MS data were analysed using the MASCOT search engine (http://www.matrixscience.com). Peptides were searched against the SWISS-PROT human sequence database and the following search parameters were employed: enzyme specificity was set to trypsin, a maximum of two missed cleavages were allowed, carbamidomethylation (Cys) was set as a fixed modification, whereas oxidation (Met), phosphorylation (Ser,Thr and Tyr) and ubiquitylation (Lys) were considered as variable modifications. Peptide and MS/MS tolerances were set to 25 parts per million (ppm) and 0.8 Da, respectively.

For the identification of phosphorylated residues, beads with bound proteins were reduced by incubating in 50 mM Tris(2-carboxyethyl)phosphine hydrochloride for 1 h at 60°C, alkylated in 200 mM methyl methanethiosulfonate for 45 min at RT, and then divided into two equal aliquots. One aliquot was digested overnight with trypsin (sequencing grade Modified Trypsin—Promega V5111) in standard conditions, while the second aliquot was digested with protease XIII (Sigma-Aldrich) in 0.1% formic acid at pH 2 for 2 h. We used this second protease because the Aurora B consensus site contains an arginine (R) or a lysine (K) upstream of the phosphorylated S or T residues ([R/K]p[S/T]). Peptide mixtures were then applied to RP-18 precolumns (nanoACQUITY Symmetry^®^ C18—Waters 186003514) using water containing 0.1% trifluoroacetic acid as mobile phase and then transferred to nano-HPLC RP-18 columns (nanoACQUITY BEH C18—Waters 186003545) using an acetonitrile gradient (5–35%) for 180 min in the presence of 0.05% formic acid with a flow rate of 250 nl min^−1^. Column outlet was directly coupled to the ion source of the Q Exactive™ Hybrid Quadrupole-Orbitrap Mass Spectrometer (Thermo Electron Corp., San Jose, CA, USA) working in the regime of data dependent on the MS to MS/MS switch. A blank run ensuring lack of cross contamination from previous samples preceded each analysis. Acquired raw data were processed by Mascot Distiller followed by Mascot Search (Matrix Science, London, UK) against the SWISS-PROT database restricted to human sequences. Search parameters for precursor and product ion mass tolerance were 20 ppm and 0.1 Da, respectively. For the enzyme specificity we selected either trypsin with one missed cleavage site allowed, or no-enzyme specificity for protease XIII digested samples. The following variable modifications were also selected: cysteine by methylthio, methionine oxidation, and phosphorylation of serine, threonine and tyrosine. Peptides with Mascot score exceeding the threshold value corresponding to less than 5% false positive rate, calculated by Mascot procedure, and with the Mascot score above 30 were considered to be positive. In addition, fragmentation spectra corresponding to the phosphorylated peptides were manually inspected.

### 4.11. Structure prediction and identification of putative Aurora B consensus phosphorylation sites

The predicted structure of CIT-K CNH_1634–1948_ was completed using the protein homology/analogy recognition engine V 2.0 (Phyre^2^) [45]. Visualization and analysis was completed using the molecular modelling software UCSF Chimera.

The GPS v. 2.1 software (http://gps.biocuckoo.org) [46,47] was used to identify consensus sites for Aurora B phosphorylation.

## Supplementary Material

Electronic Supplementary Material

## Supplementary Material

Table S1

## Data accessibility

The MS datasets supporting this article have been uploaded as part of the electronic supplementary material.
