# Supplementary material for: Cross-regulation between Aurora B and Citron kinase controls midbody architecture in cytokinesis
Source: Open Biol. 2016 Mar 23;6(3):160019. doi: 10.1098/rsob.160019 (PMC4821246; doi:10.1098/rsob.160019)
Supplement: Electronic Supplementary Material [file rsob160019supp1.pdf]

Supplementary Figures

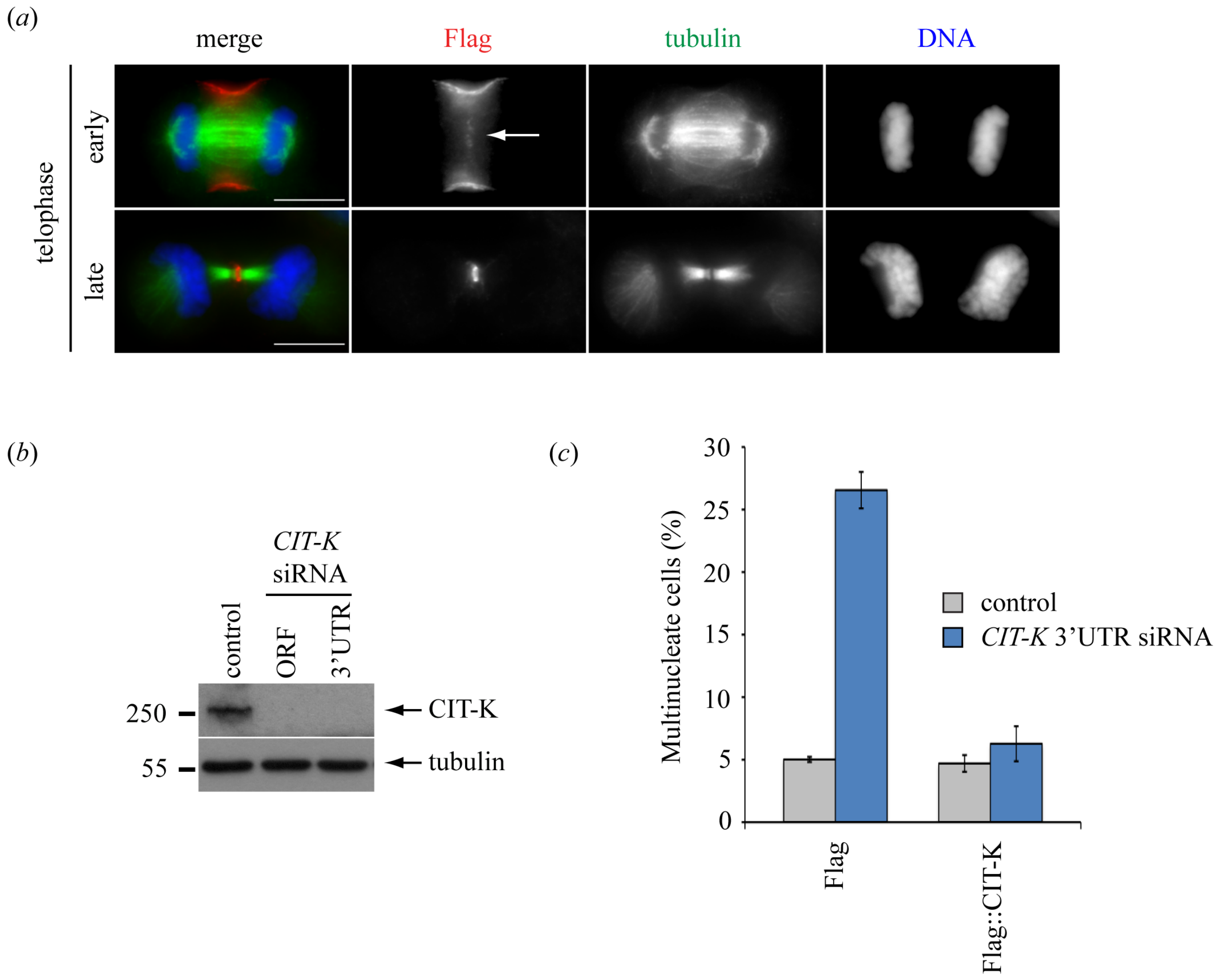

Figure S1. Characterization of the monoclonal cell line stably expressing Flag::CIT-K. (*a*) HeLa Kyoto cells stably expressing Flag::CIT-K were fixed and stained to detect Flag (red), tubulin (green), and DNA (blue). The arrow indicates CIT-K localization to the central spindle midzone. Scale bars, 10 µm. (*b*) Western blot analysis of CIT-K protein levels in HeLa Kyoto cells treated for 48 h with a scrambled (control) siRNA or siRNAs targeting either the CIT-K coding region (CIT-K ORF) or the CIT-K 3' UTR (CIT-K 3' UTR). The arrows mark the bands corresponding to CIT-K and tubulin (loading control). The numbers on the left indicate the sizes in kilodaltons of the molecular mass marker. (*c*) HeLa Kyoto cells stably expressing Flag alone or Flag::CIT-K were treated with either a scrambled (control) siRNA or a siRNA targeting the CIT-K 3' UTR (CIT-K 3' UTR) for 48 h. Cells were then stained to detect DNA and tubulin and the number of multinucleate cells was counted and plotted. Only Flag-positive cells were counted, and more than 600 cells were counted in each experiment, n=3. Bars indicate standard errors.

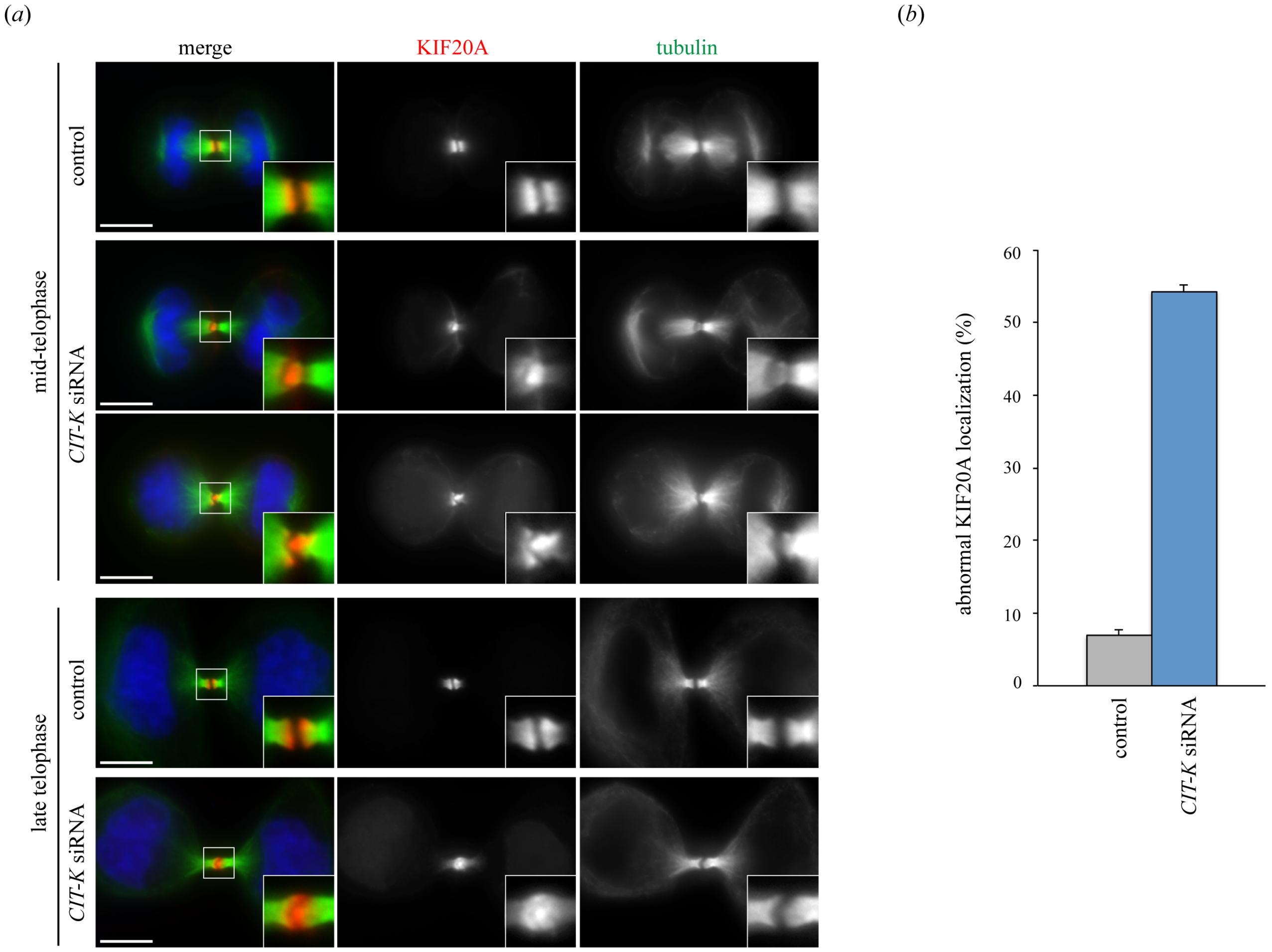

Figure S2. CIT-K is required for correct localization of KIF20A. (*a*) HeLa Kyoto cells were treated with siRNAs directed against either a random sequence (control) or *CIT-K* and after 48 hours were fixed and stained to detect DNA (blue), tubulin (green) and KIF20A (red). The insets show a 3X magnification of the midbody. Bars, 10 μm. (*b*) Quantification of KIF20A localization defects from the experiment shown in *a*. More than 100 mid-late telophase cells were counted in each experiment, n=3. Bars indicate standard errors.

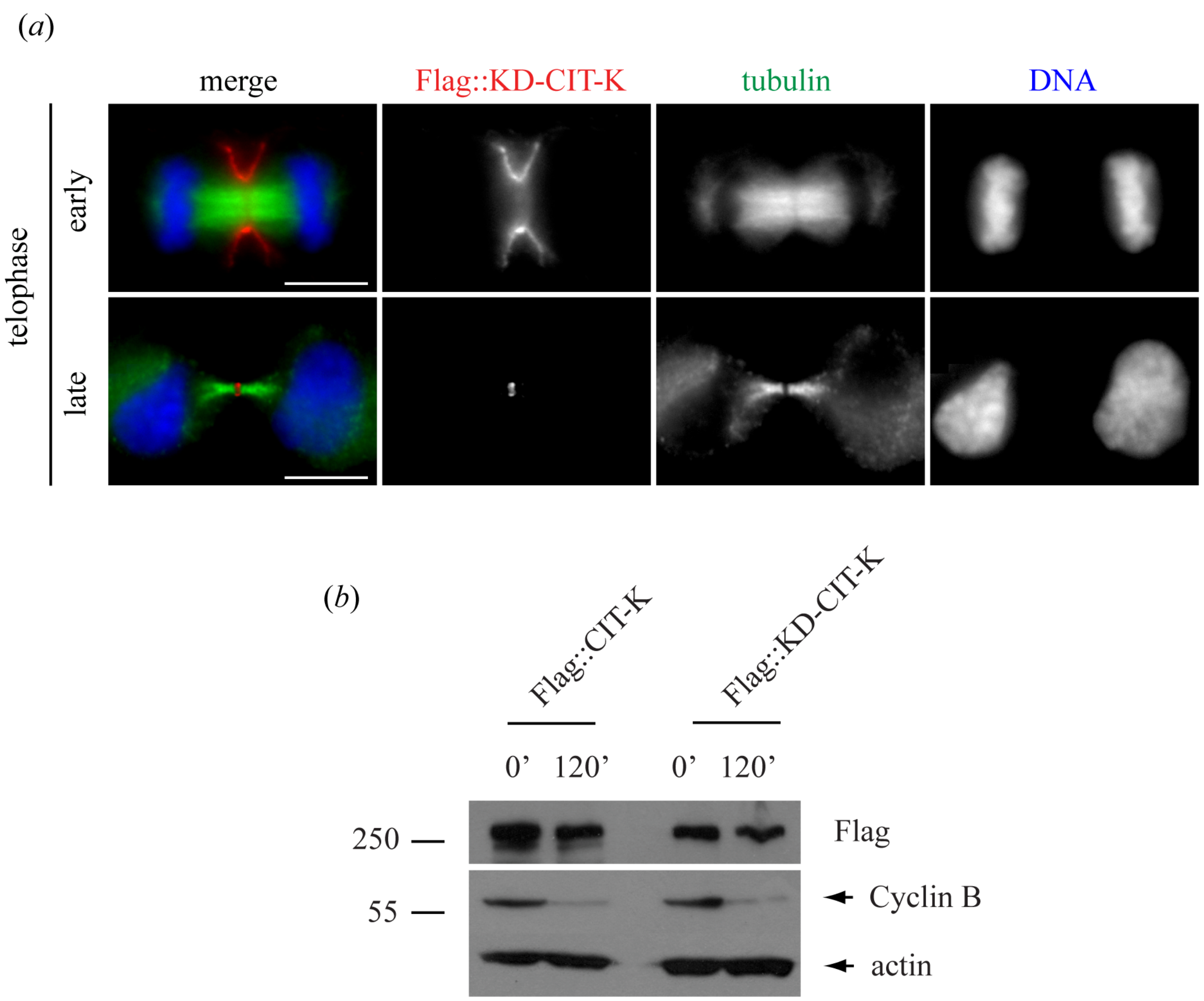

Figure S3. Characterization of the monoclonal cell line stably expressing the Flag-tagged kinase dead (KD) version of CIT-K. (*a*) HeLa Kyoto cells stably expressing Flag::KD-CIT-K were fixed and stained to detect Flag (red), tubulin (green), and DNA (blue). Scale bars, 10 μm. (*b*) HeLa cell lines stably expressing either Flag::CIT-K Flag::KD-CIT-K were blocked in metaphase by thymidine/nocodazole block and then released for 120 minutes. Proteins were extracted form cells at the time of release (0’ time point) and after 120 minutes, separated by SDS-PAGE, and analysed by Western blot to detect Flag-tagged CIT-K variants, Cyclin B, and actin (loading control). The numbers on the left indicate the sizes in kilodaltons of the molecular mass marker.

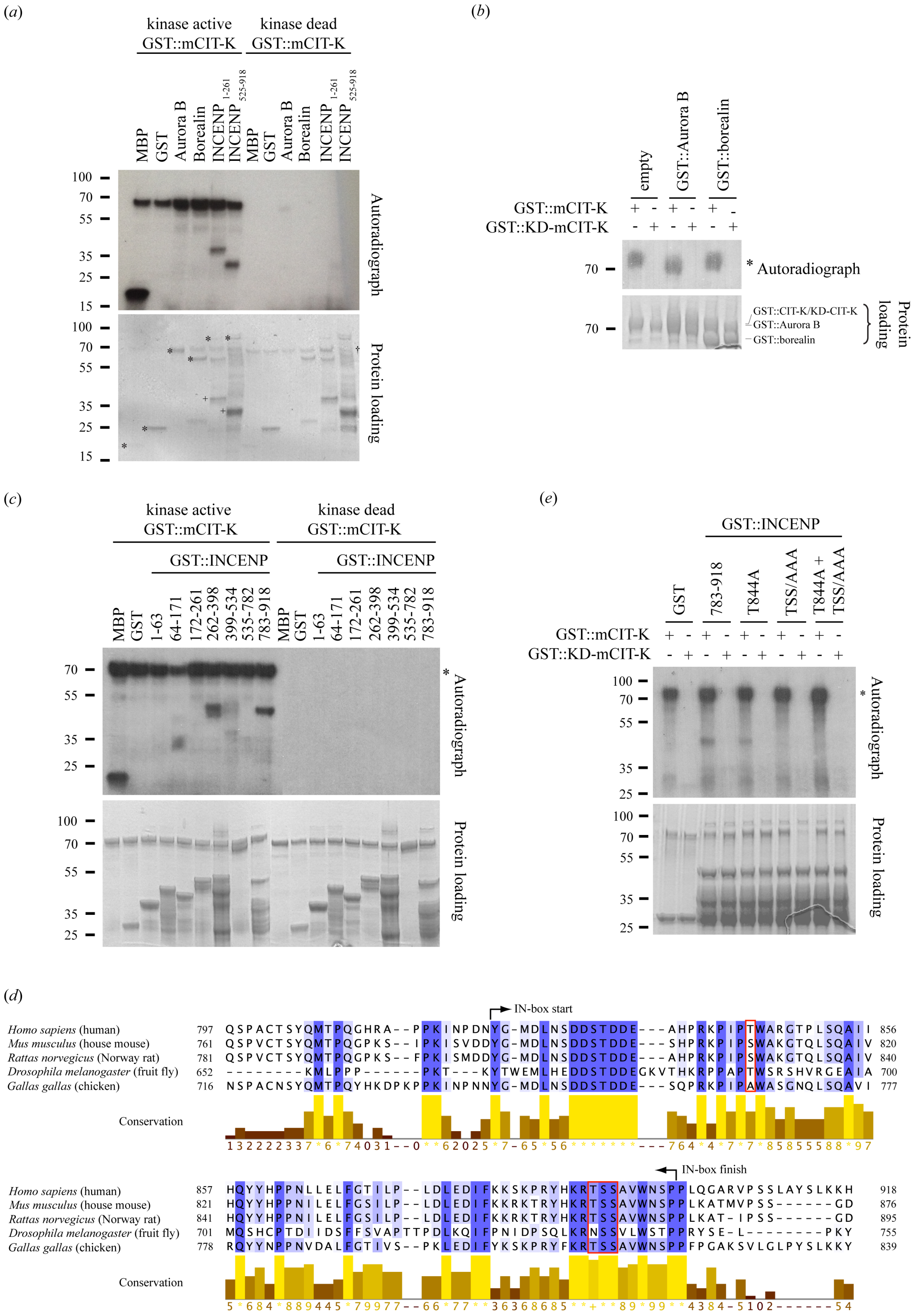

Figure S4. Mouse CIT-K phosphorylates INCENP. (*a*) GST-tagged Aurora B, borealin, N- and C-terminal INCENP, GST alone, and the positive control MBP, were incubated with GST-tagged mouse CIT-K (mCIT-K) kinase domain or KD-mCIT-K kinase domain in the presence of [γ-^{32}P] ATP. The reactions were then separated by SDS PAGE, transferred onto nitrocellulose membranes, and exposed at -80ºC. The Ponceau S staining of the protein loading is shown at the bottom. The asterisks mark the molecular positioning of the respective proteins. The cross (+) indicates protein

degradation products. The long cross (†) indicates the molecular positioning of CIT-K, which also corresponds to auto-phosphorylation on the autoradiograph. The numbers on the left indicate the sizes in kilodaltons of the molecular mass marker. (*b*) GST-tagged Aurora B and borealin were incubated with GST-tagged mCIT-K kinase domain or KD-mCIT-K kinase domain in the presence of [γ-^{32}P] ATP. The reactions were resolved by SDS-PAGE for a long period of time in an attempt to separate GST-tagged Aurora B and borealin from GST-tagged mCIT-K kinase domain. Proteins were transferred onto a nitrocellulose membrane, and exposed at -80ºC. The asterisk indicates auto-phosphorylation of CIT-K. The numbers on the left indicate the sizes in kilodaltons of the molecular mass marker. (*c*) GST-tagged INCENP polypeptides, GST alone, and the positive control MBP were incubated with GST-tagged mCIT-K kinase domain or KD-mCIT-K kinase domain in the presence of [γ-^{32}P] ATP. The reactions were then separated by SDS PAGE, transferred onto nitrocellulose membranes, and exposed at -80ºC. The protein loading is shown at the bottom. The asterisk marks CIT-K auto-phosphorylation. The numbers on the left indicate the sizes in kilodaltons of the molecular mass marker. (*d*) Amino acid sequences of the C-terminus of INCENP from different species were aligned using T-coffee (EMBL-EBI) and modified using Jalview [1], where identically matched amino acids are highlighted in darker blue. The conservation histogram (yellow and brown bars) is displayed below which highlights the level of conservation between amino acids. The red boxes highlight the two regions on INCENP that are phosphorylated by CIT-K, T844 and the TSS motif (T892, S893, S894) respectively. The arrows indicate the parameters of the IN-box, as described [2]. (*e*) GST alone, GST-tagged INCENP 783-918 and GST-tagged INCENP mutants (T844A, TSS/AAA and T844A + TSS/AAA), were incubated with GST-tagged mCIT-K kinase domain or KD-mCIT-K kinase domain in the presence of [γ-^{32}P] ATP. The reactions were then separated by SDS PAGE, transferred onto nitrocellulose membranes, and exposed at -80ºC. The protein loading is shown at the bottom. An asterisk marks CIT-K auto-phosphorylation. The numbers on the left indicate the sizes in kilodaltons of the molecular mass marker.

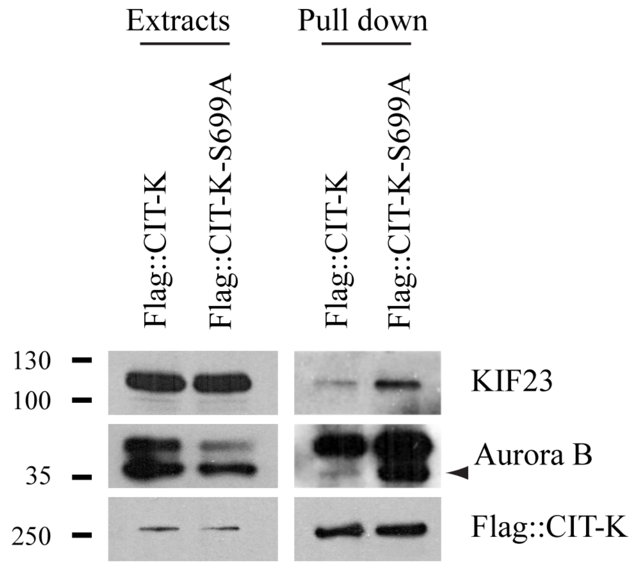

Figure S5. Flag::CIT-K and Flag::CIT-K-S699A cells bind KIF23 and Aurora B. HeLa Kyoto cells stably expressing Flag::CIT-K or Flag::CIT-K-S699A were treated with an siRNA directed against the CIT-K 3'UTR for 48 hours, blocked in metaphase by thymidine/nocodazole block, released for 90 minutes and then treated with 10 µM RO3306 for further 15 minutes. Proteins were extracted and used in a pull-down assay with anti-Flag antibodies. The extracts and pull-downs were analyzed by western blot to detect KIF23, Aurora B and Flag::CIT-K. The numbers on the left indicate the sizes in kilodaltons of the molecular mass marker.

Supplementary Tables

Table S2. List of potential Aurora phosphorylation sites in CIT-K. CIT-K sequence was analysed for peptides matching the consensus site for Aurora A and B kinases using the GPS 2.1 software (http://gps.biocuckoo.org). A cut-off score of 3.621 was applied. The position of each potential phosphorylated residue is indicated in red within its surrounding peptide sequence. The sites identified by MS in vivo are indicated in bold in the 'position' column.

Position	Residue	CIT-K region	Peptide	Score
25	S	kinase	EPIASRASRLNLFFQ	4.379
153	T	kinase	RNILSRSTSPWIPQL	4.828
445	S	CC1	LDSPAKTSSMEKKLL	5.621
**480**	S	CC1	TRLHRRVSEVEAVLS	7.517
582	S	CC1	LVSARRRSDLYESEL	7.966
592	S	CC1	YESELRESRLAAEEF	6.276
**699**	S	CC1	EAEERRHSLENKVKR	7.517
930	S	CC2	LSLQERESQLTALQA	5.828
984	S	CC2	KFDALRNSCTVITDL	3.897
1125	T	CC2	ARADQRITESRQVVE	5.138
**1385**	S	C1+PH	PSSRRKESSTPEEFS	5.552
1416	T	C1+PH	VGLNMRATKCAVCLD	4.897
1610	S	C1+PH	VVAGGRVSREKAEAD	4.034
1860	S	CNH	VDSYGRRSRTDDLKW	5.724
1898	S	CNH	VIEIQARSSAGTPAR	4.759
1899	S	CNH	IEIQARSSAGTPARA	7.552
**1962**	S	CNH	GPSTSRSSPNKRGPP	6.276

References

1. Waterhouse, A. M., Procter, J. B., Martin, D. M., Clamp, M., Barton, G. J. 2009 Jalview Version 2--a multiple sequence alignment editor and analysis workbench. *Bioinformatics*. **25**, 1189-1191. (10.1093/bioinformatics/btp033)
2. Adams, R. R., Wheatley, S. P., Gouldsworthy, A. M., Kandels-Lewis, S. E., Carmena, M., Smythe, C., Gerloff, D. L., Earnshaw, W. C. 2000 INCENP binds the Aurora-related kinase AIRK2 and is required to target it to chromosomes, the central spindle and cleavage furrow. *Curr Biol*. **10**, 1075-1078.
